# Angiotensin and Endothelin Receptor Structures With Implications for Signaling Regulation and Pharmacological Targeting

**DOI:** 10.3389/fendo.2022.880002

**Published:** 2022-04-19

**Authors:** David Speck, Gunnar Kleinau, Michal Szczepek, Dennis Kwiatkowski, Rusan Catar, Aurélie Philippe, Patrick Scheerer

**Affiliations:** ^1^ Charité – Universitätsmedizin Berlin, Corporate Member of Freie Universität Berlin and Humboldt-Universität zu Berlin, Institute of Medical Physics and Biophysics, Group Protein X-ray Crystallography and Signal Transduction, Berlin, Germany; ^2^ Department of Nephrology and Critical Care Medicine, Charité – Universitätsmedizin Berlin, Corporate Member of Freie Universität Berlin, Humboldt-Universität zu Berlin, and Berlin Institute of Health, Berlin, Germany; ^3^ Department of Nephrology and Medical Intensive Care, Charité – Universitätsmedizin Berlin, Corporate Member of Freie Universität Berlin and Humboldt-Universität zu Berlin, Berlin, Germany; ^4^ Charité – Universitätsmedizin Berlin, Corporate Member of Freie Universität Berlin and Humboldt-Universität zu Berlin, Center for Cardiovascular Research, Berlin, Germany; ^5^ DZHK (German Centre for Cardiovascular Research), Partner Site Berlin, Berlin, Germany

**Keywords:** angiotensin II type 1 receptor (AT_1_R), angiotensin II type 2 receptor (AT_2_R), endothelin type A receptor (ET_A_R), endothelin type B receptor (ET_B_R), G-protein coupled receptor (GPCR), autoantibodies, GPCR structures

## Abstract

In conjunction with the endothelin (ET) type A (ET_A_R) and type B (ET_B_R) receptors, angiotensin (AT) type 1 (AT_1_R) and type 2 (AT_2_R) receptors, are peptide-binding class A G-protein-coupled receptors (GPCRs) acting in a physiologically overlapping context. Angiotensin receptors (ATRs) are involved in regulating cell proliferation, as well as cardiovascular, renal, neurological, and endothelial functions. They are important therapeutic targets for several diseases or pathological conditions, such as hypertrophy, vascular inflammation, atherosclerosis, angiogenesis, and cancer. Endothelin receptors (ETRs) are expressed primarily in blood vessels, but also in the central nervous system or epithelial cells. They regulate blood pressure and cardiovascular homeostasis. Pathogenic conditions associated with ETR dysfunctions include cancer and pulmonary hypertension. While both receptor groups are activated by their respective peptide agonists, pathogenic autoantibodies (auto-Abs) can also activate the AT_1_R and ET_A_R accompanied by respective clinical conditions. To date, the exact mechanisms and differences in binding and receptor-activation mediated by auto-Abs as opposed to endogenous ligands are not well understood. Further, several questions regarding signaling regulation in these receptors remain open. In the last decade, several receptor structures in the apo- and ligand-bound states were determined with protein X-ray crystallography using conventional synchrotrons or X-ray Free-Electron Lasers (XFEL). These inactive and active complexes provide detailed information on ligand binding, signal induction or inhibition, as well as signal transduction, which is fundamental for understanding properties of different activity states. They are also supportive in the development of pharmacological strategies against dysfunctions at the receptors or in the associated signaling axis. Here, we summarize current structural information for the AT_1_R, AT_2_R, and ET_B_R to provide an improved molecular understanding.

## Introduction

The high biological, medical, and pharmacological relevance of GPCRs (~830 in humans) is due to their key role in signal transduction across the cell membrane from the extracellular side toward the cell interior (1). They interact with a large number of stimulants (agonists), such as odors, peptides, metabolites, light, nucleotides, amines, or a variety of hormones and proteins (2). Generally, receptor interaction with agonists results in an increased capacity of intracellular coupling and subsequent activation of G-protein(s) or arrestin(s) (3). This causes induction of downstream pathways regulating e.g., ion channel activity or gene expression (4–7). GPCR signaling is linked with almost all physiological processes, such as growth, learning, memory, reproduction, or senses like taste and vision (7). More than 100 diseases or pathogenic conditions are linked to dysfunctional GPCRs (8), including viral infections, cancer, infertility, inflammation, and metabolic and neurological disorders (9–11), which, altogether, makes these receptors essential for pharmacological and structural studies [e.g (12)]. The angiotensin (ATRs) and endothelin receptors (ETRs) belong to class A GPCRs (13, 14). For the groups of ETRs and ATRs, respectively, much detailed physiological information, but also pathophysiological relations are known.

In brief, the **AT_1_ receptor** (AT_1_R) binds different angiotensin (Ang) subtypes Ang I, Ang II, Ang III, and Ang IV, which are the main effector peptide hormones of the renin-angiotensin system (15). AT_1_R can activate the G-protein subtypes Gi/o and Gq/11, and also β-arrestin, upon agonist action (16).

Pharmacologic interventions that either decrease Ang production or modulate Ang actions through AT_1_R blockade are the current mainstay of renoprotection, as documented by extensive experimental work and clinical trials of diabetic and non-diabetic renal diseases (17). AT_1_R dysfunction leads to several pathophysiological conditions, including hypertrophy, vascular inflammation, atherosclerosis, endothelial dysfunction, insulin resistance, angiogenesis, and cancer (18). Antibodies (Abs) are involved in the development of preeclampsia, acute graft rejection, and systemic sclerosis (19–22). Of note, the Ang II/AT_1_R signaling axis was identified recently to be involved in inflammatory processes, collateral tissue damage, and systemic failure related to COVID-19 infection (23). AT_1_R blockers or biased AT_1_R agonists are discussed to contribute potentially to treatment strategies against COVID-19 effects (24–26).

Endogenous ligands of the **AT_2_ receptor** (AT_2_R) are Ang II and Ang III with affinities in the nanomolar range (14). Of note, during the elucidation of AT_2_R related signaling pathways several hypotheses arised and were studied/confirmed, including G-protein independent signal transduction (27–30), G-protein subtype Gi/o activation (31), and also ligand-independent signaling crucial in apoptosis (32). AT_2_R is expressed in vessels (endothelial cells), heart, kidney (tubules, glomeruli, collecting ducts, arterioles, and interstitial cells), brain, and immune cells (33). In the kidney, physiological stimulation of the receptor causes diuresis and natriuresis by decreasing salt and water transport from the tubules to the capillaries, triggering sodium and water excretion (34). Chronic AT_2_R overexpression has deleterious effects on cardiomyocytes (35) and AT_2_R activation, as AT_1_R, is involved in neuropathic pain (36, 37).

The **ET_A_ receptor** (ET_A_R) (38, 39) is localized mainly in vascular smooth muscle cells and, therefore, in all tissues supplied with blood, including the heart, lung, and brain, but are also present on other cell types, including myocytes within the heart (38, 40) or endothelial cells. ET_A_R has a stronger affinity for ET-1 and ET-2 than for ET-3, all three constituting the family of endothelin peptides (41). ET_A_R has been associated with the vasoconstrictive effects of ET-1 and is involved in different pathologies (6). Hence, it was shown that ET_A_R activation has detrimental effects on preeclampsia (42), heart failure (43), and pulmonary hypertension (44). In the kidney, ET_A_R induces natriuresis (45) and its inhibition can improve short-term lesions triggered by ischemia-reperfusion injury (46). Finally, point mutations in the gene coding for ET_A_R are responsible for mandibulofacial dysostosis with alopecia (47) and *Oro-Oto-Cardiac syndrome* (48), as the receptor is involved in craniofacial development. ET_A_R signaling activity is associated primarily with the G-protein subtypes Gq/11, but there are also indications for Gi/o signaling (16).

**Figure 1:**
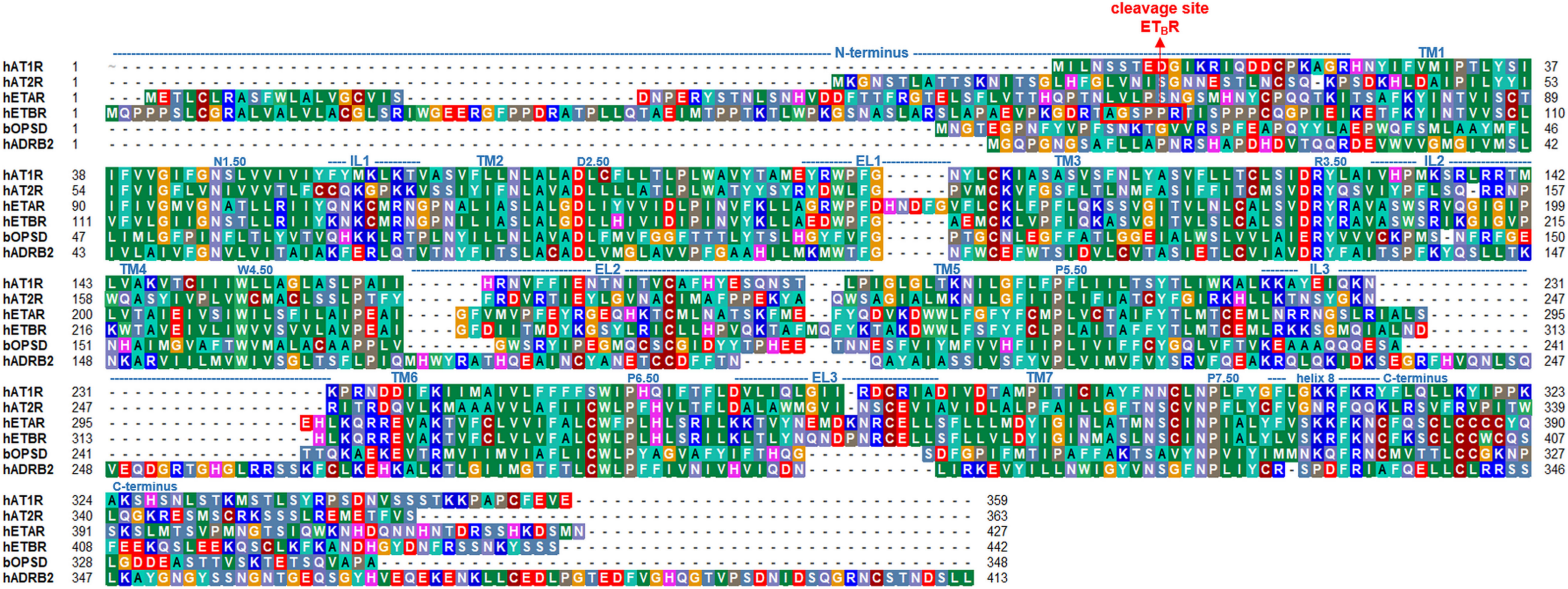
Sequence comparison between the ATRs, ETRs, and bovine rhodopsin (bOPSD) or human β-2 adrenergic receptor (hADRB2). The length of each transmembrane helix (TM1-7) or loops (IL, intracellular loop; EL, extracellular loop) are indicated above the sequence according to an AT_1_R structure [PDB ID: 4zud (53)] but can differ slightly in other structures. The overall sequence similarity between ET_A_R and ET_B_R is approximately 63%, whereas between AT_1_R and AT_2_R ~47%. Sequence similarities between ATRs and ETRs, respectively, are around 30%. The sequences of prototypical class A GPCRs bOPSD and hADRB2 are provided additionally for comparison. The alignment was visualized using the software BioEdit (54). Specific background colors reflect chemical properties of the amino acid side chains or the type of amino acid: black-proline; blue-positively charged; cyan/green-aromatic and hydrophobic; green- hydrophobic; red-negatively charged; gray-hydrophilic; dark red-cysteines; and magenta-histidine.

With the same affinity the **ET_B_ receptor** (ET_B_R) interacts with all three endothelin (ET-1, ET-2, and ET-3) peptides. It resembles many actions of ATRs on renal cell types (49). This receptor couples to the G-protein subtypes Gs, Gi/o, and Gq/11 (16). ET_B_R is expressed in the lungs and brain (50), and conveys reversal effects as ET_A_R, mainly vasodilatation by stimulating nitric oxide (NO) production and clearing ET-1 (51). In the kidney, ET_B_R is involved in sodium excretion (52). The ET_B_R contains a metal-proteinase cleavage site at the long N-terminus around an *A-G-x-P-P-R* motif (**Figure 1**) (55). Interestingly, there are reports on endothelin receptors homo- or heterodimerization with other receptors (see chapter below for details). Depending on the particular receptor-receptor configuration, the resulting signaling effects can differ (56).

In summary, AT and ET receptors are of high physiological and medical importance, including e.g., renal effects, blood pressure (57), cell proliferation (6, 58, 59), or cancer development (60). Of note, an increasing amount of structural information has been published in recent years, complementing functional insights. Several structures in different activity states were determined by protein X-ray crystallography using conventional synchrotrons or XFELs (**Table 1**) for AT_1_R, AT_2_R, and ET_B_R. They reveal details of the signal transduction process at the molecular level. In this brief review, we summarise the current state of knowledge about these receptors and receptor complex structures. We aimed to provide a first systematic overview of structural insights into these receptors including ligand binding, dimerization, receptor activation, and inactivation. Thus, we will also identify open knowledge gaps that will aid in the identification of topics relevant for future studies.

**Table 1:** Overview of ETR and ATR structures known so far (as of January 2022).

Receptor	PDB	Ligand	Modifications, fusion proteins, interaction partners	Method	Resolution (Å)	Year	References
AT_1_R	4zud	Olmesartan, inverse agonist	N-terminal BRIL; ∆1, 7-16, ∆316–59	X-ray	2.80	2015	(53)
	4yay	ZD7155, antagonist	N-terminal BRIL; ∆1, 7-16, ∆320–359	X-ray with XFEL	2.90	2015	(61)
	6do1	S1I8, angiotensin II analog, partial agonist	BRIL between 226–227; I320 to stop codon; Nb.AT110i1; dimeric receptor	X-ray	2.90	2019	(62)
	6os1	TRV023, agonist, β-arrestin bias	BRIL between 226–227; I320 to stop codon; Nb.AT110i1_le	X-ray	2.79	2020	(63)
	6os2	TRV026, agonist, β-arrestin bias	BRIL between 226–227; I320 to stop codon; Nb.AT110i1_le	X-ray	2.70	2020	(63)
	6os0	Ang II, agonist	BRIL between 226–227; I320 to stop codon; Nb.AT110i1	X-ray	2.90	2020	(63)
							
AT_2_R	5xjm	[Sar1, Ile8]Ang II, partial agonist	∆1-34 & ∆347–363; BRIL between 240–246; Fab4A03 - positive allosteric modulator	X-ray	3.20	2018	(64)
	5unf	Compound 1*, agonist	N-terminal BRIL; ∆1-34 & ∆336–363	X-ray with XFEL	2.80	2017	(65)
	5ung	Compound 1*, agonist	N-terminal BRIL; ∆1-34 & ∆336–363	X-ray with XFEL	2.80	2017	(65)
	5unh	Compound 2**, agonist	N-terminal BRIL; ∆1-34 & ∆336–363	X-ray	2.90	2017	(65)
	6jod	Ang II, agonist	N-terminal BRIL variant mbIIG between 34–35, ∆347–363; point mutation S208A; Fab4A03	X-ray	3.20	2020	(66)
							
ET_B_R	6k1q	IRL2500, inverse agonist	TEV cleavage sequence between 57–66, ∆408–442; point mutations C396A, C400A, C405A, R124Y, K270A, S342A, I381A; mT4 lysozyme between 303 & 311	X-ray	2.70	2019	(67)
	5x93	K-8794, antagonist	TEV cleavage sequence between 57–66, ∆408–442; point mutations C396A, C400A, C405A, R124Y, D154A, K270A, S342A, I381A; mT4 lysozyme between 303 & 311	X-ray	2.20	2017	(68)
	5xpr	Bosentan, antagonist	TEV cleavage sequence between 57–66, ∆408–442; point mutations C396A, C400A, C405A, R124Y, K270A, S342A, I381A; mT4 lysozyme between 303–311	X-ray	3.60	2017	(68)
	5gli	apo-state, ligand free	TEV cleavage sequence between 57–66, ∆408-442; point mutations C396A, C400A, C405A, R124Y, D154A, K270A, S342A, I381A; mT4 lysozyme between 303–311	X-ray	2.50	2016	(69)
	6igl	IRL1620 partial agonist	TEV cleavage sequence between 57-66, ∆408–442; point mutations C396A, C400A, C405A, R124Y, D154A, K270A, S342A, I381A; T4 lysozyme between 303–311	X-ray	2.70	2018	(70)
	5glh	ET-1, agonist	TEV cleavage sequence between 57–66, ∆408-442; point mutations C396A, C400A, C405A, R124Y, D154A, K270A, S342A, I381A; T4 lysozyme between 303–311	X-ray	2.80	2016	(69)
	6igk	ET-3, agonist	TEV cleavage sequence between 57–66, ∆408–442; point mutations C396A, C400A, C405A, R124Y, D154A, K270A, S342A, I381A; T4 lysozyme between 303–311	X-ray	2.00	2018	(70)
	6lry	Sarafotoxin S6b, agonist	TEV cleavage sequence between 57–66, ∆408–442; point mutations C396A, C400A, C405A, R124Y, K270A, S342A, I381A; T4 lysozyme between 303-311	X-ray	3.00	2020	(71)
ET_A_R			no 3D structures available				

Additional information is provided as the bound ligand or fusion proteins. Color code: green: active state-like; blue: inactive or antagonized; white: ligand-free.

*N-benzyl-N-(2-ethyl-4-oxo-3-{[2’-(2H-tetrazol-5-yl)[1,1’-biphenyl]-4-yl] methyl}-3,4-dihydroquinazolin-6-yl)thiophene-2-carboxamide,

**N-[(furan-2-yl)methyl]-N-(4-oxo-2-propyl-3-{[2’-(2H-tetrazol-5-yl)[1,1’- biphenyl]-4-yl]methyl}-3,4-dihydroquinazolin-6-yl)benzamide.

## Lessons From Inactive State Structures

**Figure 2:**
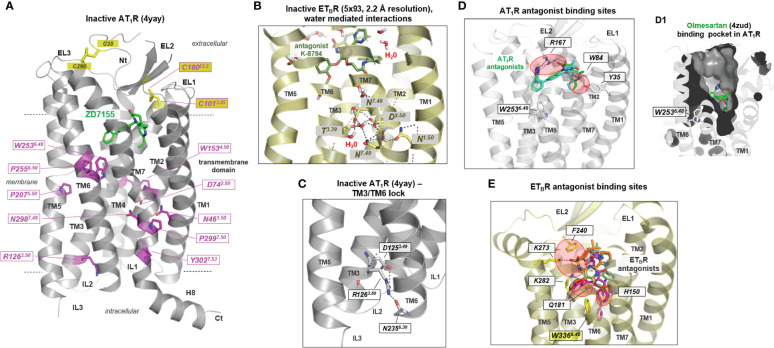
Structural features of inactive or antagonized AT_1_R and ET_B_R conformations. **(A)** Conserved residues in class A GPCRs (magenta sticks) important for receptor-fold, expression, and signaling are highlighted at the inactive state structure of AT_1_R (backbone cartoon) in complex with the antagonist ZD7155 (green sticks). Highly significant for non-active state conformations is the inward direction of the transmembrane helix (TM) 6 into the helical bundle, which closes the intracellular binding cavity for G-proteins or arrestin (see also **Figure 4E**). The antagonist ZD7155 (green) is bound in a pocket between the transmembrane helices and their transition to the extracellular loops. Notably, a disulfide bridge (yellow sticks) between the N-terminus and the EL3-TM7 transition forms and stabilizes the spatial region between the N-terminus and EL3, which is also present in the AT_2_R and the ET_B_R (not shown). **(B)** In the antagonized ET_B_R structure bound with the antagonist K-8794, water molecules solved at a high resolution of 2.2 Å. These water molecules are located centrally in the helical bundle, participating by H-bonds with hydrophilic residues in maintaining an inactive state conformation. **(C)** Of the currently known five inactive state structures for ETRs and ATRs, only one inactive state shows a H-bond between the intracellular parts of TM3 and TM6 involving the highly conserved R^3.50^. In several class A GPCRs, an “ionic lock” between this arginine and a negatively charged amino acid in TM6 has been postulated or shown to be essential for constraining the inactive state (72, 73). This cannot be perceived equally for most of the available inactive ET_B_R and AT_1_R structures. **(D)** AT_1_R antagonists olmesartan (inverse agonist, cyan sticks) and ZD7155 (green sticks, **Table 1**) are bound mainly between three residues in EL2, TM1, and TM2 in the upper part of the helical bundle (PDB IDs: 4zud and 4yay). Red circles indicate the main contact points. **(D1)** Visualized is the binding pocket of olmesartan by a clipped inner surface representation. **(E)** Superimposition of ET_B_R structures (PDB IDs: 6k1q, 5x93, 5xpr - only one backbone structure is visualized as cartoon because of high overlap between these structures) with antagonists K-8794 (green), bosentan (orange), and IRL2500 (inverse agonist, magenta) shows partially largely binding regions in the receptors, but also significant differences to antagonist binding sites of AT_1_R (red circles). While a residue of the N-terminal EL2 is involved in ligand binding in both receptors, several H-bonds to amino acids in TM3 and TM5 can be observed in the ET_B_R. The inverse agonist IRL2500 additionally contacts (blocks) the highly conserved tryptophan in TM6 (W336 in ET_B_R), which is part of the CWxP^6.50^ motif that participates in the activation mechanism of class A GPCRs. Red circles indicate the main contact points. All graphic representations in this article were created using the PyMol Molecular Graphics System Version 1.5 (Schrödinger, LLC, New York, NY). EL, extracellular loop; Nt, N terminus; IL, intracellular loop; H8, helix 8; TM1–7, transmembrane helices 1–7.

Two AT_1_R and three ET_B_R inactive state structures solved by X-ray crystallography have been published (as of January 2022; summarized in **Table 1**). They provide deeper insights into structural features associated with the inactive receptor states and how antagonists block the signaling process. Highly conserved amino acids (**Figure 2A**) significant for each GPCR class (74, 75) are generally important for expression and the folding of diverse receptor components, e.g., prolines defining weak points in helices because of steric conflicts with the preceding residue and the loss of a backbone H-bond, which can cause kinks (76, 77) as observed in the CWxP^6.50^ motif in transmembrane helix 6 (TM6) [superscripted numbers are provided additionally according to the unifying Ballesteros & Weinstein numbering for class A GPCRs (74)]. Conserved amino acids also play a fundamental role in maintaining an inactive state conformation(s), as, for example, in the AT_1_R the D74^2.50^ in the transmembrane helix (TM) 2, or N298^7.49^ in TM7 (**Figure 2A**). They interact through hydrogen bonds with each other or with other hydrophilic amino acid side chains, or with water molecules constraining the inactive state between TM’s 1, 2, 3, and 7 (**Figure 2B**). In most of the inactive state structures of AT_1_R and ET_B_R, no water or sodium ions (region between D^2.50^-N^7.49^, as known from other GPCRs (78)) can be observed due to the low resolutions between 2.7 to 3.6 Å (**Table 1**). However, in the ET_B_R structure with a resolution of 2.2 Å [Protein Data Bank (79) (PDB) ID: 5x93 (68)], water molecules in tight interaction to hydrophilic amino acid side chains are visible (**Figure 2B**). This network of hydrogen bonds between hydrophilic residues in TM1, TM3, and TM7, as well as water molecules, is not observable in all active state structures of ATRs or ET_B_R receptors, nor in other active state GPCR structures (80), because they disappear in the course of receptor activation and related structural rearrangements. Of note, in an active state, such as the ET_B_R structure complexed with the partial agonist IRL1620, a few water molecules are still observed, and they are supposed to partly preserve the interaction network typical for inactive states (70). This might be related to the fact that in this structure, as for all ET_B_R structures with bound agonists so far, no intracellular transducer protein as a G-protein molecule stabilizes the active state conformation and, therefore, the TM6 orientation is different to known fully active state structures (restricted movement toward the membrane). In conclusion, such structures do not display a fully active receptor conformation.

For diverse GPCRs a significant interaction (previously named “ionic lock”) between the highly conserved R^3.50^ in TM3 (**Figure 2A**) of the *DR^3.50^Y* motif and a negatively charged residue located at the intracellularly site of TM6 is known to be essential for maintaining the inactive state (72, 73). According to the available structures, such interaction has not yet been observed in AT_1_R or ET_B_R. Only in the case of an AT_1_R structure [PDB ID: 4yay (61)] a potential hydrogen bond interaction between R126^3.50^ and N235^6.30^ (backbone) is observable (**Figure 2C**), which may constrain the typical inactive state conformation of TM6 directed inward to the transmembrane core (**Figure 2A**) (1).

All previously known structures of inactivated or antagonized receptor states were obtained by binding antagonists (“antagonized”) or inverse agonists (“inactive”), in addition to specifically-directed mutations, which were usually necessary to stabilize an individual receptor state or improve receptor expression. (**Table 1**, **Figures 2D, E**). In the two inactive/antagonized AT_1_R structures, the ligands are bound mainly between residues located in the EL2, TM1, and TM2 (**Figure 2D**). This binding crevice (**Figure 2D1**) overlaps greatly with the binding sites of antagonists for the ET_B_R (**Figure 2E**). However, significant differences exist in binding details by an extended binding region of ET_B_R antagonists and the inverse agonist IRL2500 (**Figure 2E**). Here, specific residues in TM3 and TM5 are essentially involved in antagonist binding.

Of note, the inverse agonist IRL2500 in the inactive ET_B_R structure [PDB ID: 6k1q (67)] interacts, in addition to other residues, with an aromatic moiety directly at W336^6.48^ in TM6, which is known generally for class A GPCRs to be a crucial trigger for receptor activation. This W^6.48^ is located in the *CWxP^6.50^* motif involved in activation-related TM6 outward movement as part of the “global toggle-switch” activation model (81, 82), also described as the “rotamer toggle switch” hypothesis (1, 83). The inverse agonistic activity of this ligand is assumed to be potentially associated with this interaction, which constraints tryptophan in a basally non-active state (67). However, independent of the antagonist or an inverse agonist status, these ligands (**Figures 2D, E**) occupy a receptor region that is also involved in agonist binding (next section, **Figure 4**) and therefore compete with agonist binding.

**Figure 3:**
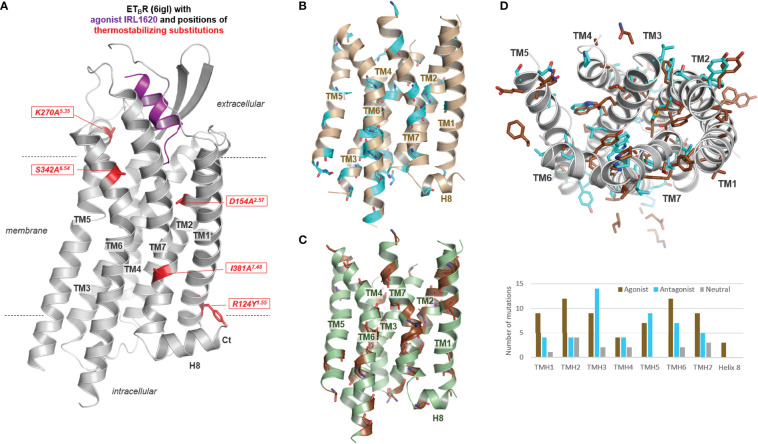
Thermostabilizing mutations of the ET_B_R and thermostabilizing mutant positions reported for class A GPCRs. **(A)** The five commonly and in combination used thermostabilizing ET_B_R substitutions are visualized at an active state structure bound with the agonist. These substitutions are also used for stabilizing inactive state and apo-state structures. **(B)** Positions (backbone as cyan-colored sticks) of thermostabilizing substitutions used for protein preparation of diverse class A GPCRs (**Supplementary Table S1**) are highlighted in a rhodopsin model (only transmembrane helices), as well as **(C)** of thermostabilizing substitutions in determined active state structures highlighted in an opsin model (brown backbone sticks). This mapping demonstrates that substitutions contributing to thermostability can be principally designed at each transmembrane helix [see also diagram in **(D)**]. **(D)** This depiction (top view from the extracellular side) of the rhodopsin/opsin wild-type side chains of positions in class A GPCRs used for thermostabilization of both states (~130 substitutions at 97 positions, **Supplementary Table S1**) demonstrates that they act in a contrasting manner, either by modulating the protein-membrane interaction or by changes of intramolecular interactions participating in the regulation of activity state-related conformations. The substitutions used for stabilizing class A GPCR structures either in the apo-, inactive-, or active-state (in the diagram termed as Agonist, Antagonist or Neutral according to the state of ligand occupancy) are located at each helix, however, more identified mutants to stabilize active than inactive state conformations are located in TM1, TM2, TM6, and TM7, whereby in TM3 and also in TM5/TM6 a high number of inactive state stabilizing mutations were identified.

Notably, aside from diverse directed structural alterations for protein stabilization such as fusion with T4 lysozyme or deletions, the inactive, apo-, and agonist bound structural complexes of the ET_B_R are modified in their amino acid sequence (**Table 1**). Five combined particular substitutions were used to stabilize complexes with both antagonists, the apo state, and also with agonists, which is not unusual in GPCR preparation for crystallization studies (**Supplementary Table S1**). These mainly alanine substitutions are located in diverse receptor regions as TM’s 1, 2, 5, 6, and 7 (**Figure 3A**). Generally, individual or combined thermostabilizing mutations used in class A GPCRs (**Supplementary Table S1**, **Figures 3B-D**) can be localized at very diverse structural parts, either with side chains directed into the transmembrane core or with side chains directed toward the membrane. A statistical analysis of the distribution of thermostabilizing mutations used for class A GPCR crystallization (analysis of 17 different GPCRs; **Supplementary Table S1** and **Figure 3D**) shows thermostabilization *via* mutations is principally feasible in each helix, including helix 8. The molecular effect of such mutations and their combinations is associated with, e.g., the stabilization of a certain conformational state (directed into the transmembrane core) as inactive or active, substitutions of residues facing lipids (directed toward the membrane or detergent), or mutations stabilizing local structural areas (e.g., helix-helix interface directed) (84, 85). In the case of the ET_B_R, a mixture of these “types” of substitutions can be postulated, whereby R124Y and I381A are directed to the membrane, D154A points into the helical core, K270A is in the interface between TM5 and EL2, and S342A is part of the TM6-TM7 interface (**Figure 3A**).

## Receptor Structures With Bound Agonists

GPCR activation commonly involves binding of an agonistic ligand or sensing of a physical trigger (e.g., light or mechanical forces), which induces alterations in the binding region and, subsequently, in specific helical adjustments relative to each other. This process finally enables intracellular binding of a transducer protein by enlargement of the crevice between the helices and ILs. The active state conformation is, therefore, stabilized by the ligand, the intracellular effector, and particular intramolecular side-chain interactions. In turn, this process, with the receptor as a central signaling hub of information, is primarily related to structural rearrangements, dependent on spatial-fit-in’s and biochemical recognition patterns [or “recognition barcodes” (86)] between the receptor-ligand complex and effector, such as the G-protein. How is this “activation process”, “signal transduction”, or “stabilization of the active state conformation” reflected by available ATR and ET_B_R structures?

**Figure 4:**
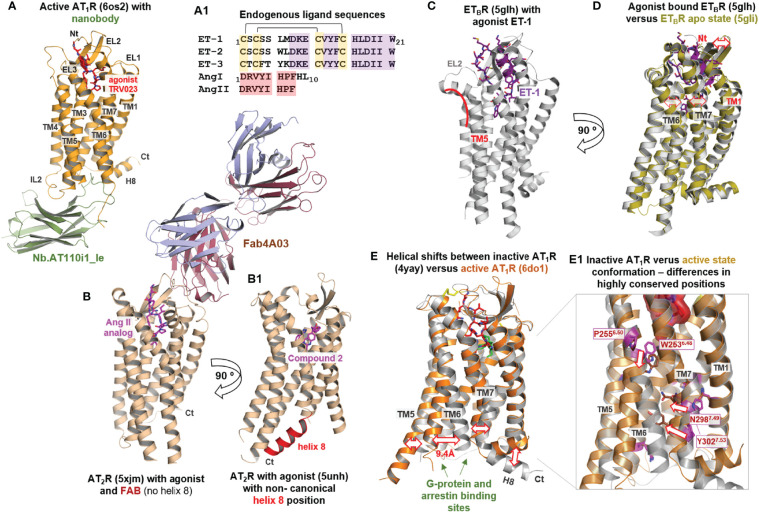
Agonist-bound and apo-state conformations of ATRs and ET_B_R. **(A)** Diverse AT_1_R structures in the agonist-bound state are already available (**Table 1**). The agonist [endogenous peptide agonist sequences are provided in **(A1)**, including annotated disulfide bridges and conserved regions (colored background)] is bound extracellularly between the ELs and their transitions to the helices (**Figure 5**). Several AT_1_R structures are stabilized intracellularly by a bound nanobody (**Table 1**). The agonist-bound structures are not complexed yet with G-protein or arrestin. **(B)** The AT_2_R structures not only contain various agonists but have been further stabilized in some cases with Fabs (fragment antigen binding), which bind on the extracellular side. **(B1)** For AT_2_R, intracellular helix 8 has been observed to be directed inward to the transmembrane helix core and stabilizes the active state structure instead of a transducer protein like the G-protein. Generally, helix 8 is oriented parallel to the membrane and outside the helical bundle in GPCRs. **(C)** The active state ET_B_R structure bound with ET-1 represents endogenous ligand binding, whereby the ligand is buried deep within the ligand-binding pocket (see **Figure 5**). The helical transition from EL2 to TM5 is kinked (red line) in contrast to the ATR structures. **(D)** Comparison with the ligand-free apo-state conformation highlights structural differences in the extracellular region where the ligand is bound, mainly in TM6 and TM7, but also for EL2 (red arrows). A further difference is the helical transition between the N-terminus and helix 1 in the apo-state structure compared to an unfolded transition in the ligand-bound structure. **(E)** Agonist-bound AT_1_R and AT_2_R receptor conformations deviate from the inactive state structures in the intracellular orientation of TM6, but also relative spatial shifts are observed at the intracellular parts of TM5 and TM7 (red arrows). For the AT_1_R, strong deviations in the H8 orientation are observable in dependency of the activity state. **(E1)** The structural transitions between inactive and active state conformations are accompanied by re-organization of intramolecular interactions in the transmembrane helical core (62), as visualized here exemplarily at amino acid residues in TM6 and TM7. This re-organization and subsequent new interactions are involved in maintaining active state-like conformations.

**Figure 5:**
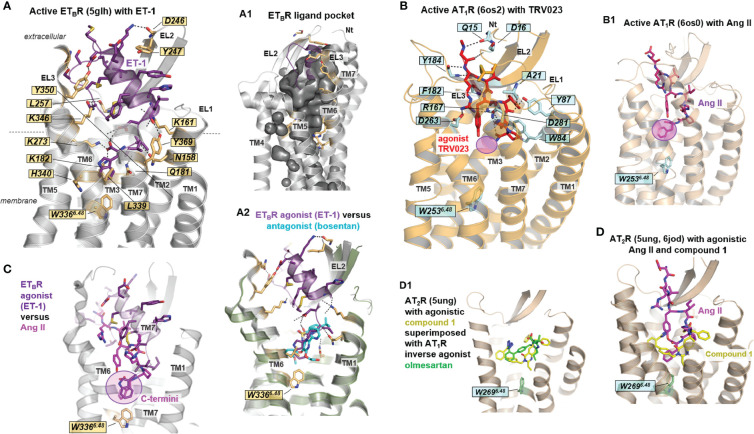
Details of agonist binding at AT_1_R, AT_2_R, and ET_B_R. **(A)** The ligands ET and Ang (or their derivatives) are bound mainly between the N-terminus, EL2, and several transmembrane helices, whereby the ligand-binding cavity is embedded deeply toward the transmembrane helical core close to tryptophan W366^6.48^ (ET_B_R). This essential tryptophan is in direct contact with the ligand-binding site of all receptors and their endogenous peptides [also **(B, B1, C)**]. Of note, the arrestin-biased angiotensin II analog TRV023 does not contact the W^6.48^ **(B-B1)** (magenta translucent circle). Many hydrophilic interactions between the receptors and the peptides can be observed, whereby four positively charged lysines and three tyrosines play a fundamental role in the corresponding ligand-receptor recognition in the ET_B_R/ET-1 complex. Generally, EL2, EL3, and the N-terminus cover the ligand-binding pocket [**(A1)**, inner surface representation] extracellularly for both ET_B_R and ATRs. Several structures reveal direct interactions between the ligand and the N-terminus, for example, in the AT_1_R- β-arrestin biased agonist TRV026 (6os2) and ET_B_R- ET-3 (6igk) complexes. **(A2)** The bound peptide ET-1 in the ET_B_R (5glh) with superimposed non-peptidic antagonist bosentan (5xpr) shows partially overlapping binding pockets close to W^6.48^. **(C)** Superimposition of ET-1 (bound to ET_B_R, 5glh) and Ang II (bound to AT_1_R, 6os0) reveal structural differences between the ligands due to deviations in sequence composition and length (see also **Figure 4A1**); however, the C-terminally located aromatic residue in both ligands is close to the highly conserved W^6.48^, which is part of the activation-related toggle switch motif in helix 6. **(D)** Non-peptide AT_2_R agonists as compound 1 (**Table 1**) are bound deep within the ligand-binding region. This section is also occupied by the endogenous peptide agonist Ang II, indicating a region highly relevant for receptor activation. **(D1)** The non-peptide inverse agonist olmesartan for AT_1_R (4zud) is principally bound in the same region as the AT_2_R non-peptide agonist compound 1 (5ung) with identical interactions to EL2. The different effects of these ligands are attributed to their detailed interactions in corresponding receptors (not visualized in detail).

More than ten ET_B_R and AT_1_R/AT_2_R structures (**Table 1**) with a bound agonist are known so far (**Figure 4**). These structures show specific features as intracellularly bound nanobodies (**Figure 3A**), extracellular bound antibody-fragments (**Figure 4B**), a non-canonical helix 8 orientation (**Figure 4B1**), or specificities in transmembrane helix conformations (**Figure 4C**). However, none of them is part of a complex with a G-protein or arrestin. However, when compared to inactive/antagonized conformations (**Figures 4E, F**), these active state-like conformations reveal how these GPCRs interact with agonists and how this binding process induces changes in receptor structure (**Figure 5**).

Generally, ATR and ETR agonists bind deep into an extracellular cleft formed between the EL1–3 and the adjacent TMs close to W^6.48^ (**Figures 4**, **5**). The EL2, EL3, and the N-terminus cover the ligand-binding pocket extracellularly for both ET_B_R and ATRs (**Figures 4A**, **5A-A1**). Receptor amino acids participating in ligand binding are located mainly at the C-terminal part of the receptor EL2, in TM2, TM6, and TM7 (**Figures 5A, B**). Further, direct interactions between the ligand and the N-terminus can be observed (AT_1_R- β-arrestin biased agonist TRV026 (PDB ID: 6os2) and ET_B_R/ET-3 (PDB ID: 6igk) complexes, **Figure 5B**).

Although no structure is available for the ET_A_R yet, it can be assumed that the binding mode of peptide-agonists at this receptor should be in principle similar to the binding mode observed at the agonist-bound ET_B_R structures. This hypothesis is based on comparison between receptor amino acids that are in direct contact to agonists (e.g. structure ET_B_R/ET-1, PDB ID: 5glh). Key contact (hydrogen bonds) amino acid residues from the receptor to the ligand are for instance K161 (TM2), K182 (TM3), E236 (TM5), R343 (TM6), K346 (TM6), Y350 (TM6), and they can be found also in the ET_A_R sequence at corresponding positions (K140, K166, E220, R326, K329, Y333). Based on this circumstance and the high overall sequence similarity of 62% between both receptor subtypes, it can be expected that the identified ET_B_R structures can serve as ideal templates to build ET_A_R homology-models. This is supported by experimental studies providing overlapping amino acids relevant for peptide-ligand binding (87). However, elucidation of potential differences in ligand binding properties (88), such as ligand affinity, definitely requires the determination of ET_A_R structures and structural complexes.

Together with W^6.48^, hydrophobic amino acids in TM3 (e.g., at positions 3.32 and 3.36) form a hydrophobic pocket that triggers receptor activation caused by endogenous ligand contact with an aromatic moiety (66). As mentioned above, this tryptophan is part of the *CWxP^6.50^* motif that participates in the activation mechanism of class A GPCRs. Superimposition of ET-1 (bound to ET_B_R, PDB ID: 5glh) and Ang II (bound to AT_1_R, PDB ID: 6os0, **Figure 5C**) reveals structural differences between the ligands due to strong diversity in their sequence composition and length (**Figure 4A1**); however, the C-terminally located aromatic residues in both ligands are close to the highly conserved W^6.48^. Of note, the arrestin-biased Ang II analog ligand TRV023 with a shorter C-terminus does not interact with W^6.48^ (**Figures 5B-B1**), indicating selective receptor activation-dependent on specific ligand features.

What else can be observed *via* a comparison of structures with agonists *vs.* antagonists? Superimposing the structure of the agonistic peptide ET-1 in ET_B_R with that of the non-peptidic antagonist bosentan reveals a partially overlapping binding mode in the vicinity of W^6.48^, indicating that this region is important for receptor activation or inhibition of activation (**Figure 5A2**). In addition, several positively charged lysines are essential for ET-1 binding to the receptor in the ET_B_R/ET-1 complex (**Figure 5A**). These lysines are also key interaction partners for antagonist binding (**Figure 2E**), suggesting the importance of the inhibitory effect of antagonists on the binding of agonists. In the case of AT_1_R, the non-peptide inverse agonist olmesartan (PDB ID: 4zud) is bound in the same region as the AT_2_R non-peptide agonist compound 1 (PDB ID: 5ung, **Figure 5D1**), including identical interactions to the EL2. The different effects of these ligands can be attributed to their detailed interactions in corresponding receptors, namely an additional hydrogen-bond of the antagonist with a tyrosine in TM1 and a contact of the agonist with W^6.48^, which is blocked by a tyrosine in TM7 (Y292^7.43^) of the AT_1_R with an inverse agonist.

Interestingly, a comparison of the ET_B_R/ET-1 complex with the ligand-free apo-state conformation (**Figure 4D**) highlights structural differences specifically in the ligand-binding region at the extracellular ends of TM6, TM7, and in the EL2. Agonist binding causes structural modifications in the extracellular part, which, is, in strong contrast to observations from the comparison between agonist-bound and inactive/antagonized structures by antagonists (**Figures 4E, E1**). The agonist-bound structures of AT_1_R and AT_2_R deviate from the inactive state structures in the intracellular orientation of TM6 (shift of ~9Å), combined with relative spatial shifts at the intracellular parts of TM5 and TM7 (**Figure 4E**). These structural transitions between inactive and active state conformations are accompanied by re-organization of intramolecular interactions in the transmembrane helical core (62) (**Figure 4E1**).

**Figure 6:**
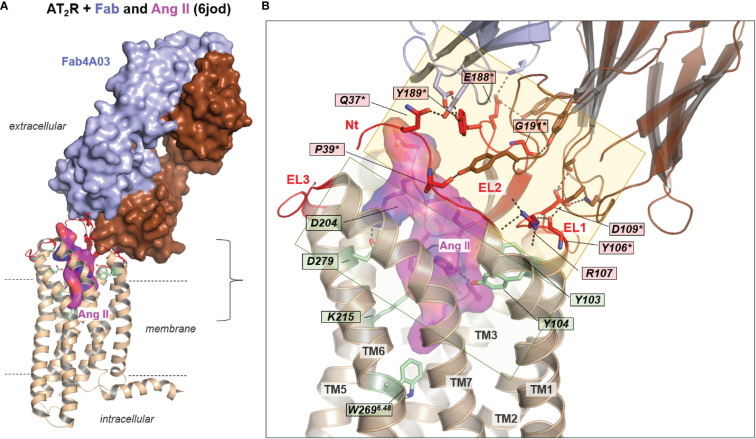
AT_2_R in complex with an antibody Fab-fragment and Ang II [PDB ID: 6jod (66)]. **(A)** The receptor is presented as a backbone cartoon, the ligand and the Fab are visualized with surfaces for clarity reasons. Amino acids involved in ligand and Fab binding are shown as sticks. Extracellular loops are colored red. **(B)** Ang II is bound deep within the helical bundle. Significant interactions can be observed with helices TM2, TM5, TM6, and EL2 (M197 backbone, R182). EL2 is involved simultaneously in Fab binding, namely with residues located in the central EL2 (E188, Y189, G191 backbone). In addition, Y106 (backbone) and D109 in the receptor EL1, contacting the Fab as well as amino acids Q37, P39 in the N-terminus. R107 in the EL1 is an important stabilizer of this constellation by several H-bonds to the transition between the N-terminus and TM1. The translucent-filled squares highlight distinguishable contact regions between the receptor with Fab and the receptor with the ligand.

As already noted, intracellular processes, such as G-protein binding or arrestin interactions concomitant to receptor-agonist complex formation, cannot yet be studied at available structures (**Table 1**). Usually, these molecules contribute toward stabilizing active state conformations. In the agonist-bound AT_1_R, a nanobody instead stabilizes the active state conformation [**Figure 4A** (63)] and, surprisingly, helix H8 is intracellularly directed inward to the transmembrane helix core of AT_2_R and stabilizes the active state receptor structure [**Figure 4B1** (65)]. This non-canonical helix 8 orientation would impede binding of G-protein or arrestin and is assumed to be related to the finding of G-protein independent AT_2_R signaling (27–30). However, in a recent AT_2_R structure complexed with Ang II a regular helix 8 orientation as known to be canonical in GPCRs is observed (PDB ID: 6jod (66), shown in **Figure 6**), which evidences that this receptor can also adapt into a conformation able to bind G-protein or arrestin.

In the agonist-bound ET_B_R structures (**Table 1**) without a nanobody, G-protein, or an inside orientated helix 8, the TM6 orientation is similar as in the inactive state conformations, whereby comparing the inactive state structure (PDB ID: 4zud) with the active state conformation (PDB ID: 6do1) of AT_1_R, a distance of intracellular TM6 of 9.4Å can be measured (**Figure 4E**). Moreover, in AT_2_R structures bound with a developed antibody Fab fragment without an intracellular stabilizer (PDB ID’s: 5xjm, 6jod), the extent of TM6 movement outside is smaller, only by approximately 7.8 Å compared to inactive AT_1_R structures, which indicates that these structures likely do not represent fully “active state conformations”.

## Antibody Binding

The available AT_2_R-Fab complexes with Ang II or its derivative [Sar1, Ile8]-AngII (64, 66) show a specific binding epitope of the Fab fragment at the receptor, which is close to the ligand ‘core’ binding region, although not overlapping. The Fab fragment (Fab4A03) acts as a positive allosteric modulator without direct interaction with the ligands but increases the affinity of both agonists (64). Such a receptor/antibody interplay is known for many GPCRs (89). Recently, a human antibody (Ab) against human ET_A_R that exhibits antitumor potency has been published (90). Autoantibodies (auto-Abs) directed against AT_1_R acting as agonists or probably positive agonistic modulators inducing pathogenic conditions have been demonstrated several times (22, 91–93) as in women with preeclampsia (21), or in patients with acute vascular graft rejection (19, 94, 95). AT_1_R auto-Abs association with clinical features has also been studied extensively in the context of transplantation (96–100), or their effects on angiogenesis in preeclampsia (101–103). Binding of activating AT_1_R-Abs promotes specific downstream signaling *via* activation of AT_1_R (19, 20); however, while Ang II binding to the receptor has been already explored intensively (104–108), the binding mode(s) between auto-Abs and receptors have not yet been determined.

Based on current literature, only AT_1_R auto-Abs from patients with transplant rejection recognize epitopes that are located primarily in EL2 (19, 21). Accordingly, the known crystallized AT_2_R-Fab complexes (64, 66) (**Table 1** and **Figure 6**) reveal that EL2 is involved in binding, namely with residues E188, Y189, and G191 located in the central EL2 (**Figure 6**). Furthermore, Y106 (backbone) and D109 in the receptor EL1 contribute to Fab binding as well as Q37 and P39 (backbone) in the N-terminus. This leads to the conclusion for ATRs that distinct receptor parts can interact simultaneously with Fabs and agonistic ligands (Figs. 4–6), whereby the concrete binding sites are distinct as at the N-terminus or EL2. This observation helps to explain how Fab fragments or antibodies mediate positive allosteric effects on signaling or directly trigger activation. The Abs may increase the predisposition of the receptor to bind Ang by a direct structural impact on the extended ligand-binding site (e.g., EL2), or/and increased signaling activity by bound Abs should lower the energetic barrier for the endogenous ligand to further stimulate the receptor. Of note, sequence comparison reveals that potential binding sites for antibodies in the EL1, EL2, and N-terminus are not conserved among ATRs and ETRs subtypes (**Figure 1**), with only a few amino acids at corresponding positions identical. This may support that so far known activating antibodies for both receptor subtypes could recognize specific structural conformations rather than binding-specific epitope residues at the receptor, which is in principle known from antibody studies at other proteins (109–111). However, different antibodies will bind naturally in a variety of ways and may differ in their receptor binding sites.

## Implications for Receptor Oligomerization and Heteromer Arrangements

The term oligomerization indicates dimeric, trimeric, tetrameric, or higher-order complexes between GPCR protomers (monomers) and has been reported for numerous GPCRs not only *in vitro* (112) but also in native tissues (*in vivo*) (113–115). Homo- or hetero-oligomerization between single receptor protomers are mostly not a prerequisite for class A GPCR signaling capacity (116), but defines the spectrum of fine-tuning options in signaling, as they can act as a functional unit (117, 118). GPCR oligomerization has been reported for several GPCR classes, such as for class A, class B, taste receptors (119–121), or class D (122).

Dimerization describes interacting xGPCR/xGPCR (homodimer) or xGPCR-yGPCR (heterodimer) constellations. For defining relevant GPCR-GPCR dimers or oligomers, several aspects are of significance, such as direct intermolecular side-chain interactions or an impact on functionalities (e.g., expression, internalization, signaling, ligand binding) compared to monomeric receptors. In heterodimerization, GPCR expression in the same cell type and cell compartment, as well as simultaneous occurrence (time-dependent expression), are prerequisites (123, 124). A large amount of GPCR-GPCR protomer interfaces with intermolecular interactions between single amino acids or between several side chains have been reported under the involvement of TM4 (125–127), TM1, and TM5-6 (128, 129). Studying the available class A GPCR dimers in determined structures, specifically the TM1-TM1/helix8-helix8 and the TM4-TM4/TM5-TM5 interfaces, occur often (130). However, different oligomer GPCR interfaces for homo- and heterodimers can be assumed, whereby likely no universal interface exists. Supposedly, receptor interfaces are of dynamic character (131) and GPCRs are expressed as a mixture of monomers and homomers, whereby the two forms may interconvert dynamically (132). Several examples demonstrate that GPCR oligomerization can have a major impact on the signaling properties of interacting protomers, e.g., in ligand binding (133, 134), G-protein coupling specificity, and signal transduction mechanisms (114), or cell surface expression (135). In the event of a direct mutual effect of GPCRs organized in dimeric arrangements, a horizontal allosteric impact on each other, either positively or negatively, may occur (136).

For the ATRs and ETRs, a tremendous set of information is available, supporting a wide spectrum of oligomer formations. As exemplarily summarized from literature databases and a direct collection of GPCR oligomers (GPCR Interaction Network, http://www.gpcr-hetnet.com (137)), the following oligomers have been reported for ATRs or ETRs:

- *AT_1_R* with PAR1 (138), μOR (139), prostaglandin F2aR (140), ET_B_R (141), RXFP1 (*in vivo* (142, 143)), ADRB2 (144), AT_2_R (145), CB_1_R (146), secretin receptor (SCTR, class B) (147), bradykinin B_2_R (148);
- *AT_2_R* with AT_2_R (149), bradykinin B_2_R (150);
- *ET_B_R* with D_3_R (151), ET_A_R (56, 152–154); and
- *ET_A_R* with µOR (155).

Oligomerization of wild-type and a non-functional AT_1_R mutant inhibits Gαq-mediated signaling but not ERK activation, supporting a functional influence of a homo-oligomerization (156). Aldosterone-related effects activate AT_1_R and AT_2_R hetero-dimerizations (149), altering trafficking and arrestin recruitment profiles (145). Further functional effects reported to be associated with homo- or heterodimerization are, for example, transactivation and synergism [AT_1_R with PAR1 (138)], altered expression levels for AT_1_R - ET_B_R heteromers (141), or ATRs with RXFP1 show functional crosstalk in myofibroblasts (142, 143). AT_2_R heterodimerization with bradykinin B_2_R (150) has a strong impact on the signaling outcome and amplitude (NO production). ET_B_R-ET_A_R heterodimers are modified in internalization rates compared to the homo-dimerization of the wild-type receptors (152).

**Figure 7:**
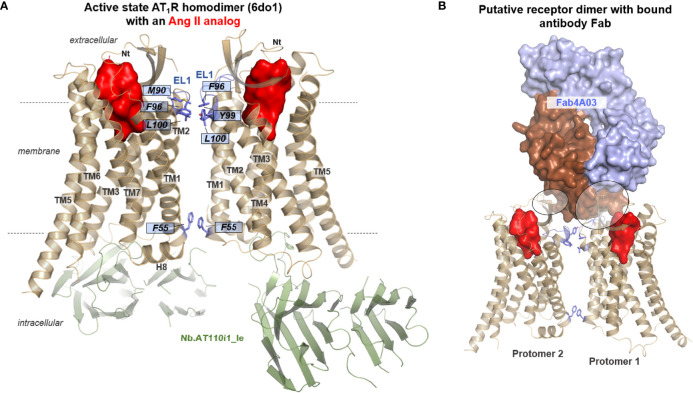
Dimer arrangement of the active state AT_1_R bound with an Ang II analog and nanobodies. **(A)** The complex between the Ang II analog, AT_1_R, and active state stabilizing nanobodies has been crystallized as a homodimer [PDB ID: 6do1 (62)]. The interface between the protomers is constituted by hydrophobic and aromatic amino acid side chain contacts at EL1 (M90, F96), TM1 (F55, intracellularly), TM2 (Y99), and helix 3 (L100). **(B)** In a putative scenario of a dimeric receptor arrangement with antibody binding at one protomer, the Fab fragment should also simultaneously contact the second receptor protomer. For this model, the AT_2_R structure (6jod), with and without a Fab, were arranged together as suggested by the AT_1_R homodimer.

To date, only one report on the AT_1_R homodimer structure exists [PDB ID: 6do1 (62)]. The interface between the single protomers is constituted by hydrophobic and aromatic amino acid side chain contacts at EL1, TM1, TM2, TM3, and helix 8 (**Figure 7A**). Interestingly, this dimer is in an active state conformation, bound with an Ang II analog and with intracellularly stabilizing nanobodies at each protomer. The observable interface in the AT_1_R dimer is in agreement with interfaces in many other GPCR dimers (157), which might imply relevance also *in vivo* to cause a mutually allosteric (158) functional impact on ligand binding capacities or internalization rates. However, other interfaces were studied and recently proposed by atom molecular dynamics simulations (159), which is in line with the assumed multitude of feasible GPCR oligomer arrangements.

As exemplified in **Figure 6B** in a dimeric receptor formation, a bound antibody at one protomer should simultaneously contact the second protomer (**Figure 7B**). This should be the case for homodimers of AT_1_R (156), AT_2_R (149), or heterodimers of ATRs (145) and ETRs (56, 141), which are known to be occupied endogenously by antibodies under pathogenic conditions (160, 161). As already mentioned above, an AT_2_R/Ang II analog complex was co-crystallized with a Fab. This Fab acts as a positive allosteric modulator (64), which might also be related to observed dimeric receptor constellations or might have consequences on the functional reactivity of receptor dimers.

Finally, if homo- or heterodimeric ATR and ETR arrangements are of functional and physiological relevance, pharmacological interventions may (must) target or consider these oligomers, especially with the aim of circumventing adverse effects mediated by allosteric heterodimer actions. Correspondingly, if the large number of putative heterodimers between ATRs/ETRs and other GPCRs are functionally relevant *in vivo*, any pharmacological intervention at their interaction partner should also have an impact on both receptor subtypes (ETR, ATR), which might be registered medically as unwanted adverse effects. Pharmacological strategies may profit from homo- or heterobivalent ligands specifically entering GPCR dimers (162, 163) in diverse ligand constellations, e.g., as bitopic and dualsteric ligands (164).

## Concluding Remarks

As summarized in this short review, an enormous amount of structural-functional information on ATRs and ETRs is available, with a clear boost on structure determination since 2015. These structures provide details and general insights into mechanisms of activation and features of nonactive or inactive states. An advantage of the high number of solved structures is the resulting capability for comparison, including diversities in ligand binding, and to study the spectrum of possibilities in structural arrangements, e.g., helix conformations or dimer formation. However, several gaps in knowledge are evident, with primary emphasis on not yet determined ET_A_R structures and missing structural information on G-protein or arrestin binding. Moreover, reflecting the high number of GPCR heteromer reports for ATRs and ETRs with functional impact, it also appears necessary to intensify further means of exploring ways to elucidate heteromer arrangements, both structurally and functionally for these receptors and binding partners. In addition, this is an area of utmost pharmacological importance (165, 166) and, therefore, must be of structural interest, especially given the increasing possibilities in the determination of complex structures (167). Finally, the relevance of autoantibody binding to both receptor groups require questions on antibody binding and its functional significance to be explored in-depth, intending to use improved understanding to tailor the design of optimal ligands useful for pharmacological intervention strategies or to recruit these receptors (as monomers or dimers) as hubs for precisely sought specific responses.

## Author Contributions

Manuscript writing: DS, GK, and PS. Figure and table preparation: DS and GK. Manuscript editing: MS, DK, RC, and AP. Data analyses: DS, GK, DK, and PS. Supervising: GK and PS Funding: PS. All authors contributed to the article and approved the submitted version.

## Acknowledgments

This work was supported by the Deutsche Forschungsgemeinschaft (DFG) through CRC 1365, – Project-ID 394046635 – SFB 1365, subproject A03 (to PS); through CRC 1423, project number 421152132 – SFB 1423, subprojects A01 (to PS); and through the European Union’s Horizon 2020 MSCA Program under grant agreement 956314 [ALLODD] (to PS).

## Conflict of Interest

The authors declare that the research was conducted in the absence of any commercial or financial relationships that could be construed as a potential conflict of interest.

## Publisher’s Note

All claims expressed in this article are solely those of the authors and do not necessarily represent those of their affiliated organizations, or those of the publisher, the editors and the reviewers. Any product that may be evaluated in this article, or claim that may be made by its manufacturer, is not guaranteed or endorsed by the publisher.
